# Hepatology through the crystal ball

**DOI:** 10.1007/s12072-019-09959-y

**Published:** 2019-07-04

**Authors:** Roger Williams

**Affiliations:** 1 The Institute of Hepatology London, Foundation for Liver Research, 111 Coldharbour Lane, London, SE5 9NT UK; 2 Faculty of Life Sciences and Medicine, King’s College London, London, UK

**Keywords:** Alcohol, Obesity, Gut dysbiosis

## Abstract

The rapidity of the increase in the global burden of liver disease covered in this review with estimates worldwide of 2 million deaths from cirrhosis and with no signs of effective controls being introduced for two of the main causes, namely, excess alcohol consumption and obesity, is of great concern. The 25% prevalence of non-alcoholic fatty liver disease in many population groups and the recent description of primary hepatocellular cancer (HCC) in obese subjects without underlying severe fibrosis/cirrhosis also raises many questions. In addition, covered in this review are more encouraging areas including techniques for machine preservation of donor organs enabling previously marginal organs to be used for transplantation. Greater knowledge of gut microbiome and gut bacterial translocation is defining the inflammatory reaction underlying multi-organ failure in decompensated cirrhosis. The gut microbiome also influences the response of HCC patients to the new check-point inhibitor drugs which restore immunological responses of its host.

## The global burden

With Asia and Pacific having the highest number of deaths from liver disease and India alone accounting for one-fifth of all cirrhosis deaths globally, it is not an inappropriate time to gaze into the crystal ball at how this might be changed over the next 5–10 years. Worldwide, the estimates are of 2 million deaths from cirrhosis and 1 million from viral hepatitis and primary hepatocellular carcinoma (HCC). 1.3 billion adults are overweight with a 25% prevalence of NAFLD. Figures for chronic viral hepatitis are equally staggering with hepatitis C virus (HCV) affecting 71 million subjects, including 5.6 million people who inject drugs (PWID), 2.6 HIV infected with an additional 1.75 million new infections annually. Chronic HBV infections are even higher at 252 million worldwide [1]. As for alcohol-related liver disease, two large epidemiological assessments published over recent months (Global Burden of Disease Study) [2] and (Global Statistics Report on Alcohol Health) [3] describe a ‘globalisation’ of alcohol-related harm over the coming decades with a continuing increase in overall consumption and there is evidence in that at least in females, there are no safe levels of alcohol consumption [4].

**Fig. 1:**
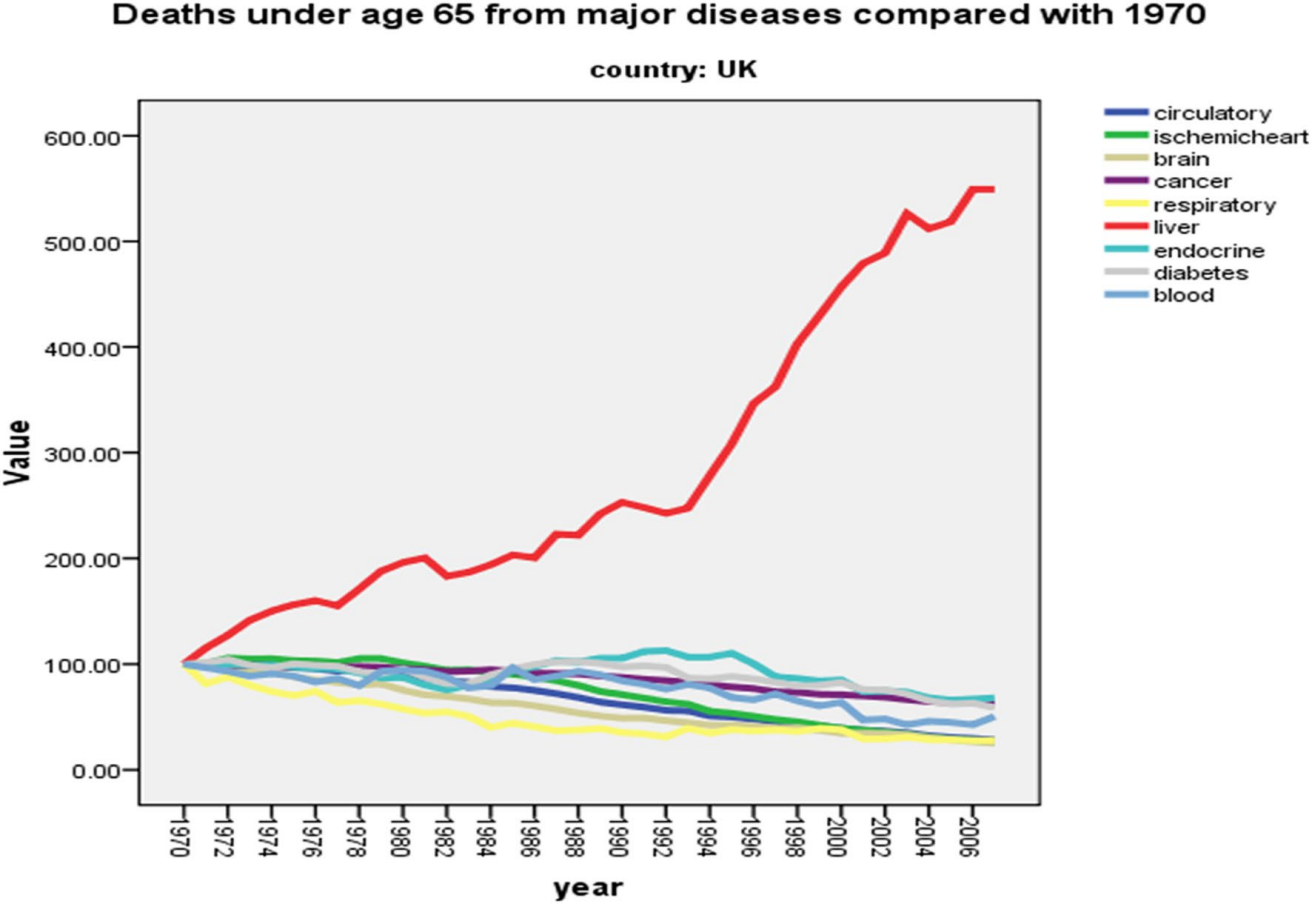
Percentage changes in standardized UK mortality rates (age 0–64 years) normalized to 100% in 1980. Standardised Mortality Rate data for the UK were downloaded from the World Health Organisation Health for All Database (http://data.euro.who.int/hfadb/) and normalized to 100% in 1980. (Analyzed by Nick Sheron September, 2013)

Although the lessening of HCV-related disease with the new directly acting antiviral agents (DAAs) is encouraging and there is promise ahead for HBV, the chances of major reductions in disease burden from lifestyle causes of alcohol and obesity are not great. Indeed, my experience in leading the Lancet Commission into Liver Disease in the UK over the past 5 years has shown just how difficult it is to get effective policy and government action in the lifestyle area. The Commission was set up to address the steadily rising standardized mortality rate for liver disease in the country compared with falling rates for other chronic disorders—strokes, COPD and cardiovascular events (Fig. 1) [5], and it is not too difficult to understand why this was happening. The publication by the Academy of Medical Sciences in 2004 appropriately entitled “Calling Time” showed the inverse correlation between consumption and the falling price of alcohol [6] and increasing affordability, as well as greater access is likely to be relevant in other countries worldwide.

## Work of Lancet Commission into liver disease in the UK

But despite all the hard evidence amassed by the Commission [7] including the massive financial savings in healthcare costs that would accrue, the UK Government have so far been unwilling to intervene on the basis that how much people drank was a personal responsibility and that the introduction of fiscal measures would be highly unpopular with the electorate—a view strongly championed by the powerful industry drinks lobby who have also been effective in resisting the proper labeling of alcohol products. A recent survey showed inadequate labeling of the number of units and appropriate health warnings were virtually non-existent. Health warnings were highly effective in the campaigns for reducing smoking and the majority of the public support mandatory labeling of alcohol products [8].

Two fiscal measures have proven effectiveness, namely, the Minimum Unit Price (MUP) policy which sets a level (usually 50 pence) below which a unit of alcohol cannot be sold. This targets particularly the heavy drinkers who are dependent on buying cheap alcohol and a tax escalator on alcohol duty directed at those who though not in the highest risk category are nevertheless drinking at hazardous levels. But perhaps there is the beginning of some movement here. An MUP policy was introduced into Scotland in May 2018, after the Supreme Court refuted finally the appeals of the Scottish Whisky Association, which over 5 years had blocked the Scottish Government in introducing the policy. It is to be implemented in Wales next year and plans are being considered in Northern Ireland. It will be interesting to see how this is tackled by the new European Association for the Study of the Liver (EASL) Lancet Commission into Liver Disease, with the widely differing cultural, economic and political structures of the European countries. In the low-income countries, attending to underlying social deprivation will also be of critical importance.

As to the disease burden from high levels of obesity namely NAFLD, NASH, cirrhosis and primary HCC, numerous studies have shown significant interaction, indeed synergies, with alcohol consumption. In a large-cohort study from Scotland, a BMI > 35 doubled the risk of alcoholic liver disease for any given intake of alcohol [9] and a Finnish 2000 cohort found synergistic interactions between alcohol and multiple components of the metabolic syndrome [10], with diabetes particularly a major risk factor for the development of cirrhosis. Obesity has been shown to increase the rate of progression of cirrhosis whatever the etiology, underlying mechanisms relating to an inflammatory reaction in biologically active adipose tissue.

## Rising incidence of primary hepatocellular carcinoma

The number of HCC cases worldwide has increased by 75% since 1990 with NAFLD the most rapidly rising cause. Chronic HBV and alcohol account for one third of the cases and HCV infection 21%. The recent reports of NAFLD patients presenting with HCC without underlying cirrhosis or even severe fibrosis are particularly worryingly and raise difficult questions as to appropriate screening regimes [11]. On a positive note, the imposition of a levy by the UK Government on sugar content in soft drinks, as part of a strategy for controlling childhood obesity produced half the forecasted revenue for the exchequer as a result of industry reformulating their products and was reported in the media as “that’s good news”. The levy is comprised of a charge of 18 pence per liter and 24 pence per liter for two sugar bands of 5 g and 8 g/100 ml, respectively. A similar measure for reducing the harmful content of sugar, fat and calories of packaged food would be expected to be similarly effective.

## Eradication of hepatitis C viral infection

Doubts are being expressed as to whether WHO’s 2030 target for eradication of HCV infection is achievable with the difficulties in accessing groups such as drug addicts. Although recently published series of PWID’s treated by DAAs report a marked decrease in viremia prevalence and low reinfection rates [12]. Even if infection is not completely eradicated with the DAAs, the number of infected cases and disease burden resulting will be greatly reduced. A large VA cohort of infected HCV subjects treated in the USA showed a 72% reduction in mortality from end-stage cirrhosis and a 58% decrease in the number of HCC cases [13]. The reduced number of cases needing transplantation according to the European Liver Transplant Registry resulted in approximately 600 more donor organs being available for treating new or expanded disease indications [14]. In fact, the slack has already been taken up by an increase in numbers being transplanted for NASH and alcoholic liver disease [15]. Indications already figuring more in transplant selection are those with decompensated cirrhosis and multi-organ failure (acute-on-chronic liver failure—ACLF). In one recent series, the survival rate for ACLF patients with three organ failures at 90 days was 78%, compared with 5.9% for those treated medically [16].

The wider use of machine perfusion for improving function of marginal organs should also enable an increase in transplant numbers. Only 63% of UK deceased donors are transplanted largely on account of steatosis of the donor organ which will increase likelihood of ischemic and reperfusion injury to a graft [17]. Ex situ machine preservation either highly oxygenated at 10 °C or the more simple normothermic perfusion has been shown to decrease occurrence of graft injury and gives excellent 5 year graft survivals (Table 1) [18].

**Table 1:** Machine perfusion for marginal donors

16.6% of retrieved livers 2015–2016 NOT transplanted with steatosis main reason (39%) for declining
Steatosis increases ischemic injury during cold storage and enhances reperfusion injury
Ex situ machine preservation
Highly oxygenated at 10 °C or normothermic at 37 °C, (± defatting agents) decreases steatosis and improves metabolic profile

Finally, in looking into the crystal ball, it is likely that a new area of translational research, namely, the fecal microbiome will be featuring in a number of disease areas. Gut dysbiosis in cirrhosis with intestinal translocation of bacteria from the gut to the liver is thought to enhance the damaging inflammatory reaction and the occurrence of multi-organ failure. How far it will be possible to influence disease progression through correcting gut dysbiosis by a fecal transplant or oral probiotics remains to be determined. A successful liver transplant brings back the normal diversity in gut flora and is correlated with an improvement in the patient’s cognitive function [19]. The fecal microbiome also influences clinical responses to the use of check-point inhibitor drugs such as nivolumab in the treatment of HCC which can restore the host’s immunological responses to tumor tissue. Elegant experiments have shown that a fecal microbiome transplant from a responding case will restore immune reactivity in a mouse model of HCC [20].
